# Photocatalytic Treatments for Personal Protective Equipment: Experimental Microbiological Investigations and Perspectives for the Enhancement of Antimicrobial Activity by Micrometric TiO_2_

**DOI:** 10.3390/ijerph18168662

**Published:** 2021-08-16

**Authors:** Lory Marika Margarucci, Gianluca Gianfranceschi, Vincenzo Romano Spica, Giuseppe D’Ermo, Cristiano Refi, Maurizio Podico, Matteo Vitali, Ferdinando Romano, Federica Valeriani

**Affiliations:** 1 Department of Movement, Human, and Health Sciences, Laboratory of Epidemiology and Biotechnologies, University of Rome “Foro Italico”, 00135 Rome, Italy; l.margarucci@studenti.uniroma4.it (L.M.M.); gianluca.gianfranceschi@uniroma4.it (G.G.); federica.valeriani@uniroma4.it (F.V.); 2 Department of Surgery “P. Valdoni”, Sapienza University of Rome, 00185 Rome, Italy; giuseppe.dermo@uniroma1.it; 3 GeneS, Research Start Up, 00187 Rome, Italy; refi@genes4you.it; 4 Department of Chemistry, Life Sciences and Environmental Sustainability, University of Parma, 43124 Parma, Italy; maurizio.podico@unipr.it; 5 Department of Public Health and Infectious Diseases, Sapienza University of Rome, 00185 Rome, Italy; matteo.vitali@uniroma1.it (M.V.); ferdinando.romano@uniroma1.it (F.R.)

**Keywords:** titanium dioxide, photocatalysis, disinfection, mask, nanoparticles, PPE

## Abstract

The COVID-19 pandemic has led to countries enforcing the use of facial masks to prevent contagion. However, acquisition, reuse, and disposal of personal protective equipment (PPE) has generated problems, in regard to the safety of individuals and environmental sustainability. Effective strategies to reprocess and disinfect PPE are needed to improve the efficacy and durability of this equipment and to reduce waste load. Thus, the addition of photocatalytic materials to these materials, combined with light exposure at specific wavelengths, may represent promising solutions. To this aim, we prepared a series of masks by depositing micrometer-sized TiO_2_ on the external surfaces; the masks were then contaminated with droplets of bacteria suspensions and the coatings were activated by light radiation at different wavelengths. A significant reduction in the microbial load (over 90%, *p* < 0.01) was observed using both Gram negative (*E. coli*) and Gram positive (*S. aureus*) bacteria within 15 min of irradiation, with UV or visible light, including sunlight or artificial sources. Our results support the need for further investigations on self-disinfecting masks and other disposable PPE, which could positively impact (i) the safety of operators/workers, and (ii) environmental sustainability in different occupational or recreational settings.

## 1. Introduction

Filtering facepiece (FFP) masks are a type of personal protective equipment (PPE) aimed to preserve one’s respiratory system against contaminated air droplets or suspended particles [1]. The use of face masks has been recommended in community settings (by different health authorities and in several countries) [2]. For example, a healthy individual could wear a mask as protection when in contact with an infected individual, while an infected individual could wear a mask to prevent transmission of a virus [1,2,3]. There are different masks with different sizes, shapes, and properties. They are classified into three categories: (i) cloth face mask, (ii) surgical face mask, and (iii) filter facepiece respirators (FFRs); each are equipped with filters, including N95, P100, FFP2, FFP3, and KN95, complying with International Standards [4,5,6,7]. Several factors can influence the quality and filtering abilities of face masks, affecting their efficiency, such as the size and shape of particles, rate, pattern of flow, charge state of particles, breath frequency, humidity and temperature, and usage time [8,9,10,11,12,13,14,15,16,17,18,19]. Potential contamination of the outer surface of FFPs entails an infection risk for the user (including “ordinary” individuals, health operators, or workers employed in different sectors) [20]. Moreover, prolonged use and the accumulation of external contaminants can reduce the filtration efficiency, further transforming these devices into pathogen receptacles and/or vehicles. To limit this risk, the mask must be replaced periodically (consequently increasing non-recyclable waste that must be disposed of properly) [20,21]. The COVID-19 pandemic has exacerbated all of these aspects; the world has witnessed increased infection risks, enforced preventive measures, and a reduction in the availability of protective masks/PPE. This scenario revealed the need for alternative solutions, by implementing safe and effective reuse of PPE, while reducing the disposal load [22,23,24]. One promising strategy is based on decontamination procedures that would not damage the different filter layers of the mask or impair their functionality [25,26]. To this aim, several approaches were proposed: steam sterilization, hydrogen peroxide vapors, gamma, ultraviolet and microwave irradiation, ethylene oxide, moist heat incubation, dry heat treatment, autoclave treatment, and chlorine-based solutions [27,28,29,30,31,32,33,34]. Recently, several innovative strategies (i.e., light-activated photosensitizer materials or heavy metals) were proposed to disinfect surfaces [35]. The use of “covering surfaces” containing silver, copper, and zinc, are regulated by the European Union Biocidal Products Regulation (BPR, Regulation (EU) 528/2012) and by Regulation (EC) no. 1907/2006 of the European Parliament, and of the Council on the Registration, Evaluation, Authorization and Restriction of Chemicals (REACH) [36]. Furthermore, antimicrobial nanocomposites based on Titanium Dioxide (TiO_2_) have been actively investigated [37,38,39,40,41]. The mechanism of TiO_2_ activity on microorganisms can be outlined by the production of reactive oxygen species (ROS), with cell wall damage and lipid peroxidation of the cell membrane caused by particle-cell attachment by electrostatic force, and the oxidation of many organic constituents of the microorganism, such as protein alteration and/or DNA damage [42]. Titanium Dioxide (titanium (IV) oxide or Titania) is a naturally occurring oxide of titanium. It is generally used as a dye (Pigment White 6—PW6- or CI 77891), independently of the catalytic potentials. This compound, sourced from ilmenite, rutile and anatase, has a wide range of applications, including paint, sunscreen, and even widely diffused food coloring, identified by code E171. Several studies have investigated the toxicity of TiO_2_ as a food additive, and the International Agency for Research on Cancer (IARC) has classified TiO_2_ pigment nanoparticles as a potential carcinogenic factor of group 2B (likely carcinogenic to humans) based on tests on animals regarding exposure by inhalation [43,44,45]. The debate is ongoing; the European Food Safety Authority (EFSA), based on all of the available evidence, stated that a concern for genotoxicity cannot be excluded. Given the numerous uncertainties, the panel concluded that E171 can no longer be considered safe when used as a food additive [46,47]. However, TiO_2_ is largely used for a variety of personal care products, including sunscreen and cosmetics [48,49,50]. Indeed, TiO_2_ is considered skin-friendly material and apparently cannot penetrate the deeper layers of the skin [48,49,50,51,52,53]. Thus, TiO_2_ has substantial advantages over chemicals (NO, H_2_O_2_, small organic molecules) and metal (e.g., Ag-based systems). TiO_2_ nanoparticles (NP) showed a broad spectrum of activity against Gram-negative and -positive bacteria and fungi, acting against multiple drug resistant strains. Moreover, titanium dioxide-polymer nanocomposites were considered environmentally safe, exerting a non-contact biocidal action [54,55,56,57,58]. One main critical issue in the risk–benefits ratio related to the specific antimicrobial use of this photocatalytic material concerns the particle size, in particular the nanometric profile [49]. The study of antimicrobial activities induced by micrometric particles (MP), or even larger, and the introduction of pioneering strategies for fixing NP or MP on different surfaces may be promising alternatives [53]. Optimization of light-activated photosensitizer materials and their coatings on different matrices can open up new perspectives to fulfill the antimicrobial needs for masks and PPE. In order to overcome the limits related to the nanometric threshold and to explore the alternative potential use of TiO_2_ MP-coated materials in PPE, the TiO_2_ MP antimicrobial activity was tested on cloth face masks, surgical masks, and FFP2 masks.

## 2. Materials and Methods

### 2.1. Design of Study

**Figure 1:**
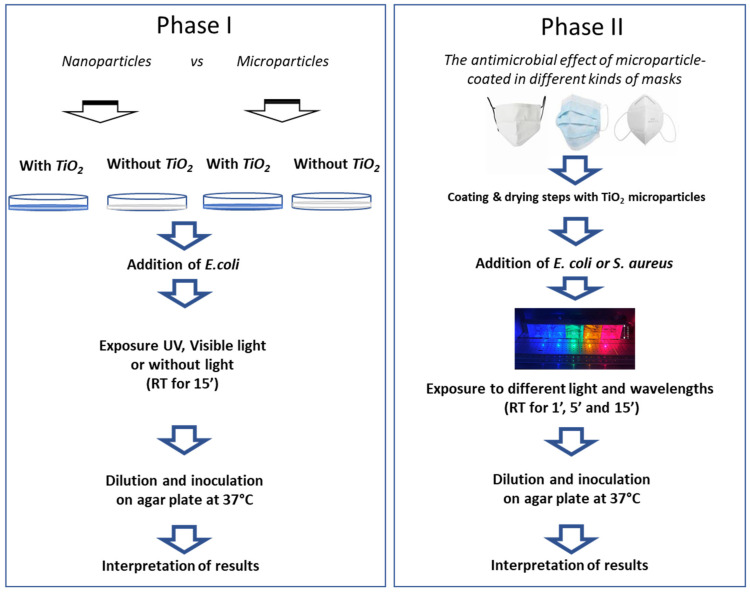
Experimental set-up. The study involved two main steps: (**I**) preliminary investigation of antimicrobial effects of microparticles with respect to nanoparticles exposed under several light wavelengths (control was without TiO_2_ particles); and (**II**) testing the antimicrobial effects of different microparticle-coated masks (cloth face mask, cloth surgical mask, and FFP2 mask). This flowchart summarizes the approach used to test the antibacterial activity of light on TiO_2_-coated masks.

The study was divided into two phases (Figure 1): (i) the investigation of antimicrobial effects of TiO_2_ microparticles with respect to TiO_2_ nanoparticles under several light wavelengths; (ii) the antimicrobial effects of microparticle-coated masks: cloth face masks, surgical face masks, and FFP2 masks.

**Figure 2:**
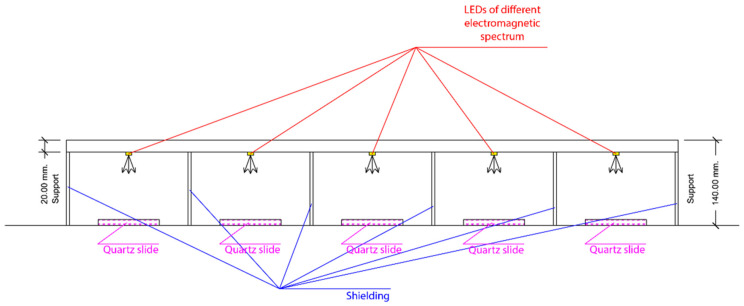
**Rainbow exposure system**. A dedicated devise was applied to perform, in parallel, simultaneous light treatments under identical conditions, but using LED with different wavelength emissions. The exposure setting ranged from 350 to 630 nm, and temperature was monitored in real time by a thermocouple thermometer. Distance from the light source and the target was constant for all exposure chambers. To avoid evaporation, a quartz cover was only considered for longer exposures when using water solutions.

In order to perform parallel exposures under different light wavelengths, a specific device was developed (Figure 2).

### 2.2. Bacterial Strains and Culture Conditions

Gram-negative *Escherichia coli* (ATCC 25922) and a Gram-positive *Staphylococcus aureus* (ATCC 25923) strains were used to test the photocatalytic antibacterial effects. The strains were cultured on Tryptic Soy Agar (Oxoid, Basingstoke, UK) and incubated aerobically at 37 °C overnight.

### 2.3. Materials

Three different kinds of masks were selected for this study: (i) a cloth face mask (80 % polyamide and 20 % elastane); (ii) surgical masks (comply with EN 14683); and (iii) FFP2 (CE 2233). Nanoparticle (18 to 24 nm and 1.0 ± 0.1 % *w*/*w* of concentration) and microparticle (1–4 μm and 1.2 ± 0.1 % *w*/*w* of concentration) suspensions in water were obtained from different producers (Sigma Aldrich, Saint Louis, MO, USA; Chem Spec srl, Peschiera Borromeo, Italy) and their stability was guaranteed at temperatures ranging from 5 °C to 30 °C. Anatase TiO_2_ nanoparticles (pH 5.1) were synthesized by the sol–gel method, while for microparticles, Rutile TiO_2_ (pH 7) was micronized in distilled water solution. Each suspension was diluted 1-fold with the distilled water and no further components were present.

### 2.4. Exposure

Exposures at different time points were performed using a dedicated LED-based display system (Figure 2), encompassing a UV source and light sources in the visible spectrum. The exposure system was set up; it included 5 LEDs (5 W of power) at the following wavelengths: 395–400 nm, 450–455 nm, 515–525 nm, 590–595 nm, and 620–630 nm, as previously described [55]. This Rainbow System was improved and optimized to achieve consistent exposure conditions under different light wavelengths. The used setting considered light emission ranging from 395 to 630 nm, in independent single exposure chambers isolated by aluminum shields to avoid light contaminations and open from the front side to assure identical environmental parameters and external temperature conditions. Samples were located below the LED system at 14.0 ± 0.5 cm. The temperature of the bacterial suspension during the illumination was monitored using a Fluke 54 thermocouple thermometer (Everett, Washington, DC, USA) at one-minute intervals to exclude major warming differences and thermic effects. As additional controls, the same bacterial cells (treated and not treated) were used, protected by light (in a box or aluminum foil wrapped), in parallel, under the same conditions of the exposed ones. Additional exposures were tested under common everyday outdoor and indoor conditions (data not shown), performed under sunlight (Lat 41.31712, Long 12.457429, CET Time: h 13–14), indoor light (neon 3.5–4 W of power at a distance of 80 cm), and a white light LED lamp (395–630 nm distance 15 cm, 5 W of power). During the experimental tests, the temperature variations ranged from 22 °C to 31 °C.

### 2.5. Phase I: Comparison between Nano (NP)- and Micro-Particles (MP) of TiO_2_ in Sterile Water Medium

An established model based on the *E. coli* bacteria strain was used, following previously reported protocols [55,56,57]. Briefly, a bacterial suspension was prepared from frozen aliquots of the same stock of *E. coli* ATCC 25922. Aliquots were rehydrated into fresh LB medium (5% *v*/*v* for *E. coli*) and incubated overnight at 37 °C until the bacteria reached the stationary growth phase. After incubation, the absorbance of the suspension was measured at 600 nm to determine the bacterial concentration, according to calibration curves (DS-11 Series Spectrophotometer-Fluorometer, DeNovix Inc., Wilmington, DE, USA). Therefore, bacterial cells from the same master culture were diluted (about 1:1) in sterile water to obtain the final bacterial suspension to perform the experiments (1.0 ± 0.2 × 10^9^ CFU/mL). For comparison between nano (NP)- and micro-particles (MP) of TiO_2_, 14 mL of suspensions containing 1.4 mL of 10^9^ cells/mL of *E. coli* master culture and 12.6 mL of suspensions of TiO_2_ were prepared. The NP and MP-TiO_2_ stock solution was diluted to 0.1% wt. The suspension of NP–TiO^2^ used for this experiment was at a concentration of 0.1 ± 0.1 % *w*/*w*, while the suspension of microparticles was at a concentration of 0.1 ± 0.1 % *w*/*w*. The suspension without TiO_2_ was prepared with 1.4 mL of master culture and 12.6 mL of distilled water. The 14 mL of described suspensions were transferred into two 60 mm Petri dish plates (7 mL for each one), and independently exposed at room temperature at different wavelengths, without the lid during exposure. Simultaneously, control samples from the same master culture were incubated for each wavelength in parallel under identical conditions, but in absence of TiO_2_. Furthermore, 2 samples with TiO_2_ and 2 without, were incubated in the dark for the same time as the treatment groups (15 min). After each dose of light had been delivered, 50 µL aliquots were withdrawn and streaked on TSA agar plates and CFU counted after overnight incubation at 37 °C.

### 2.6. Phase II: Antimicrobial Effect of Microparticle-Coated Masks

Microparticle suspensions tested in phase I were used to coat three kinds of masks and verify the antimicrobial effects on their surfaces.

#### 2.6.1. Coating of Mask

All masks were cut into pieces of about 7 × 5 cm, and wetted only on one side with a suspension of TiO_2_ microparticles, at concentrations of 1.2 ± 0.1%*w*/*w*, dried in a microbiological hood at room temperature for about 24 h. 

#### 2.6.2. Preparation of Test

An overnight bacteria suspension (10^9^ CFU/mL) was diluted to 10^4^ CFU/mL in sterile water. To each mask type (treated and not treated)—3 spots, with diameters of 0.5 mm by 2 µL of the suspension 10^4^ CFU/mL, were applied. The bacterial concentration of each spot at Time 0 (T_0_) was evaluated, seeding serial dilution aliquots of the starting suspension in order to estimate the number of CFUs per microliter of the applied suspension. Coated masks were exposed (described in Section 2.4) for 1, 5, and 15 min. At the end of the exposure, each piece of tissue was removed with sterile tweezers and placed on a selective growth medium to test microorganism growth [59], using Brilliance™ *E. coli*/coliform selective agar (Oxoid, Detroit, MI, USA) for *Escherichia coli* and Mannitol Salt Agar (Oxoid, USA) for *Staphylococcus aureus.* The plates were incubated at 37 ± 1 °C for 18–24 h. The number of bacterial concentrations, at 1, 5, and 15 min, were determined, counting bacterial colonies present in the agar plate, and the number of CFU was reported as CFU/mL. The percentage of surviving bacteria was calculated with respect the T_0_ microbial load.

### 2.7. Statistical Analysis

Each treatment and mask, including the control, was conducted (at least) in duplicate and the results are presented as mean ± SD (standard deviation). For each sample, data were normalized with respect to the reference control without TiO_2_. Student’s *t*-test was used for pairwise comparisons. Statistical analyses were performed using the SPSS 22.0. (SPSS for Windows; SPSS Inc., IBM, Chicago, IL, USA) at a significance level <0.05.

## 3. Results

**Figure 3:**
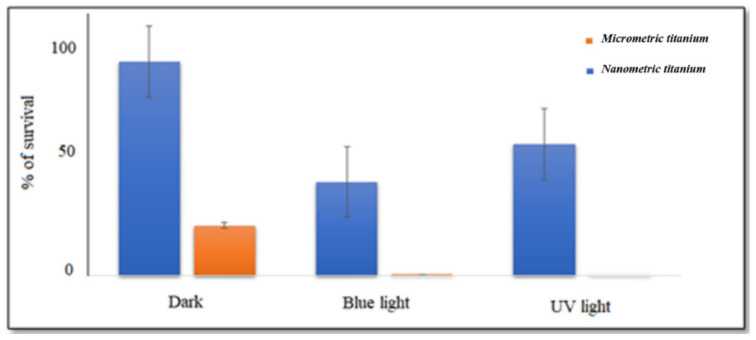
**Antimicrobial activity of micrometric and nanometric TiO_2_ in water.** UV and blue light irradiation trigger the antimicrobial potential of TiO_2_ microparticles, showing an even higher efficiency with respect to nanometric particles. Comparison at different wavelengths (dark, blue light at 450–455 nm and UV-light at 395–400 nm) in water using *E. coli* as a model after 15-min exposure.

The first series of experiments were conducted in water suitably contaminated by *E. coli*, to avoid confounding factors related to the different matrices or coating methods. We used both TiO_2_ MPs and NPs, which showed both bactericidal properties. This effect was observed at UV frequency and blue light frequency, in particular within the spectrum of 400–470 nm, which is already known to have intrinsic antimicrobial properties [55,58]. A higher effect was shown for MPs compared to NPs, and the percentage of *E. coli* reduction within 15 min reached 100% when exposure was performed under UV-light at 395–400 nm, and over 98% when using blue light at 450–455 nm (Figure 3).

**Figure 4:**
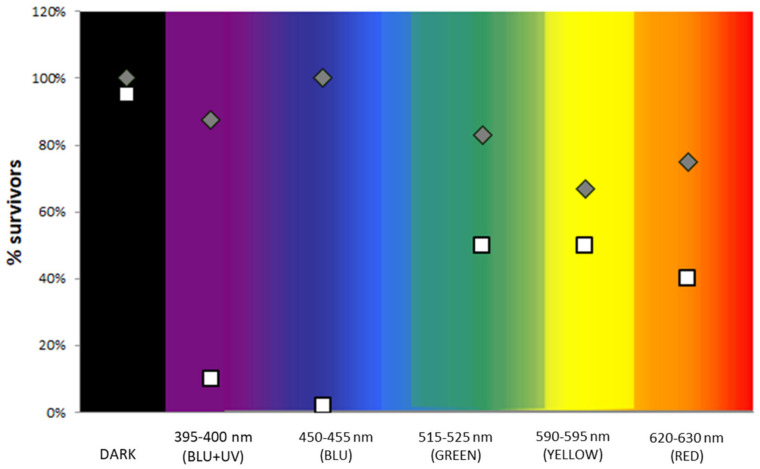
**Photocatalytic effectiveness of micrometric TiO_2_ coated masks at different wavelengths.** Experimental contamination of cloth face masks, previously with coated (white square) or without coated (grey rhomb) TiO_2_ microparticles. Data are expressed as the percentage of survival on agar plates, after 15-min exposures at several wavelengths of the visible spectrum, and using dark (not light exposure) as a control. UV (395–400 nm) and blue light wavelength (450–455 nm) show the highest antibacterial activity (98–100%).

Based on the data obtained from the experiments carried out in water, the second series of experiments, performed in phase 2 of the study, only evaluated the action of TiO_2_ MPs coating without testing NP coatings. In particular, the TiO_2_ MPs were coated on pieces of cloth face masks and irradiated at different wavelengths, ranging from 395 to 630 nm. A 98–100% decrease in the number of CFU/mL was obtained when wavelengths at 395–400 nm and at 450–455 nm (blue light) were used (Figure 4). Similar results were observed for the other types of masks, both for the Gram-negative *E. coli* and for the Gram-positive *S. aureus*.

**Figure 5:**
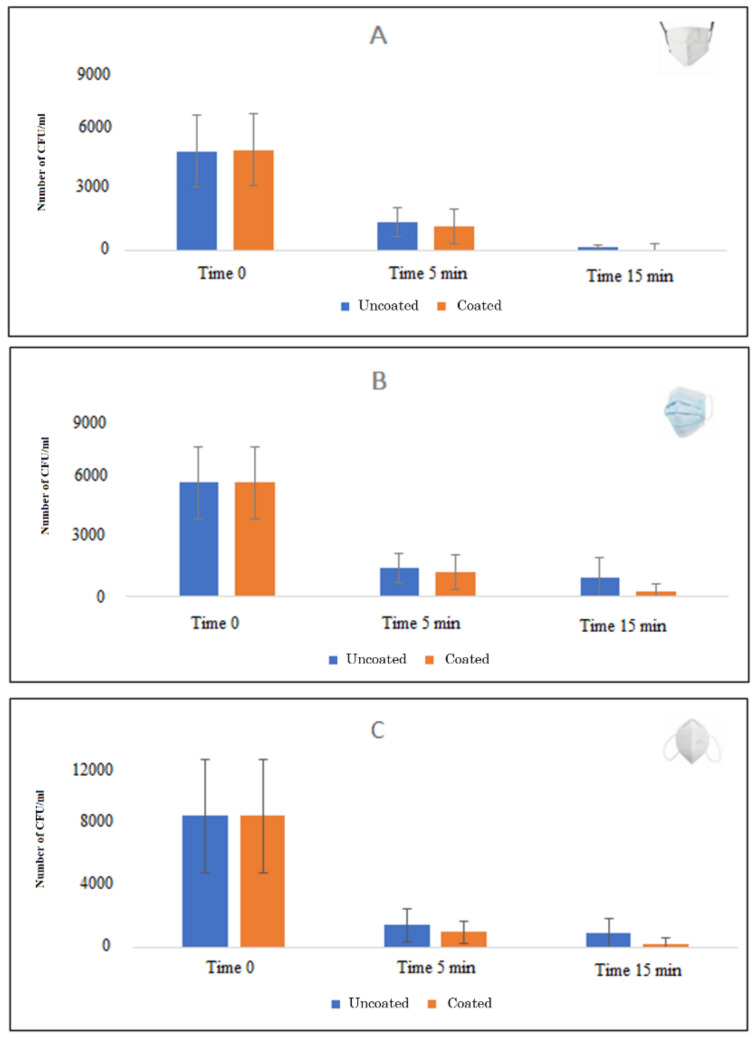
**Blue light antibacterial activity on different masks.** TiO_2_ treatment shows enhancement of the antibacterial activity on different mask materials. Quantification of *E. col*i bacterial cell viability at different times after blue light exposure (Time 0, Time 5 min, and Time 15 min) with (coated) and without TiO_2_-coating (uncoated). CFU: colony forming units. (**A**): cloth face mask; (**B**): surgical mask; (**C**): FFP2 mask.

Table 1 summarizes the results obtained after exposure to blue light for *E. coli* and *S. aureus*. In all TiO_2_ coated masks, the percentage of CFU/mL reduction achieved values higher than 90%, as shown in Figure 5.

**Figure 6:**
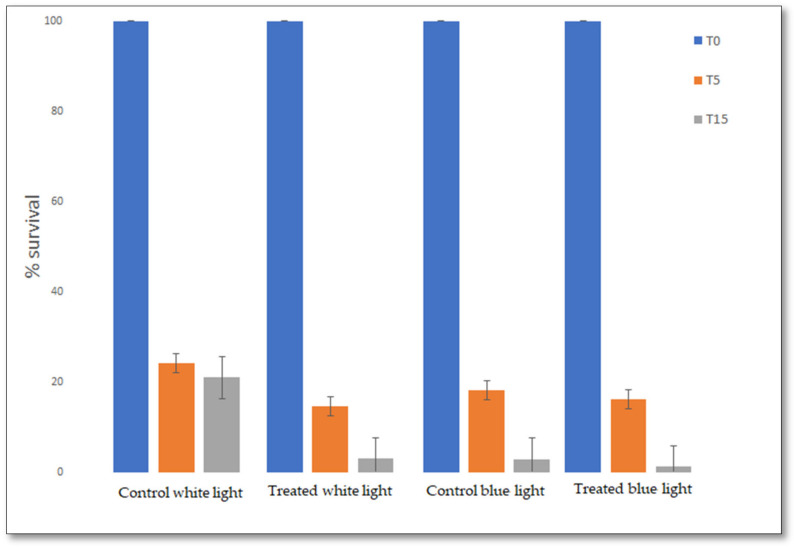
**The effect of TiO_2_-coating, independently of mask type.** The means of all observations obtained with coated or not coated-TiO_2_ masks are shown as percentages of survival (*E. coli* CFU/mL) at time 0 (T_0_), 5 min (T_5_), and 15 (T_15_) minutes of exposure of white-visible and blue light. The combination of visible light irradiation and TiO_2_-coated show an enhancement of the antimicrobial activity, suggesting that the coating of TiO_2_ can be useful with visible light irradiation and independently of the type of mask.

The mean values of all exposed samples are shown in Figure 6, suggesting the possibility of obtaining an antibacterial photocatalytic effect independently from the kind of material used for the mask and its FFP level. The exposure with blue light (nm = 450–455) induced a significant increase in CFU/mL reduction (>98%, *p* = 0.0151), somehow overcoming the action of the TiO_2_ MPs. Otherwise, the enhancement of the antibacterial effect obtained by TiO_2_ treatment was more evident, even with no selected wavelength when using white artificial light exposure, which shows different wavelengths within a wider range of frequencies (Figure 6). Indeed, after 15-min exposure under LED-generated white light, an over 95% CFU/mL reduction of viable *E. coli* was detected, further supporting a specific role for TiO_2_ coating. Similar experiments were performed under natural sunlight, with comparable enhancement of the antibacterial effect (data not shown).

**Figure 7:**
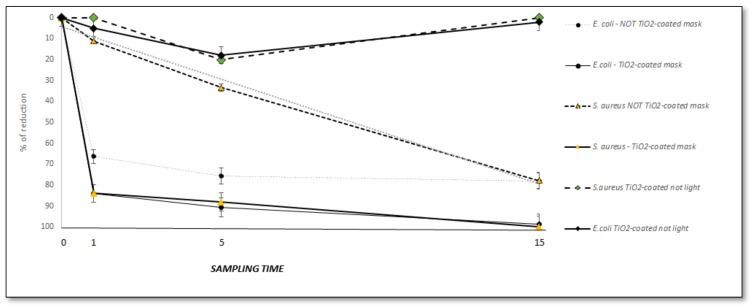
The reduction trend of *E. coli* and *S. aureus* in the FFP2 mask coated by TiO_2_. The percentage of the reduction of *E. coli* and *S. aureus* as a function of the times of exposure at blue light are reported. The level of *E. coli* and *S. aureus* decreases with increasing times of exposure at blue light, but a significant enhancement of the antimicrobial activity was observed when exposing coated-TiO_2_ masks. Sampling times were 0, 5, and 15 min.

Finally, we tested the enhanced antimicrobial effect of TiO_2_ under blue light at shorter exposure times. Figure 7 presents the CFU/mL reduction of *E. coli* and *S. aureus* as a function of the time of exposure for TiO_2_-coated or not-coated FFP2 masks.

The effect of blue light on TiO_2_-coated FFP2 mask increased, showing a reduction of 84% within 1 min for both the Gram negative and Gram-positive bacteria (*E. coli* R_2_ = 0.900, and *S. aureus* R_2_ = 0.900). Interestingly, in absence of TiO_2_-coating, the time required to reach the reduction of 70% in CFU/mL exceeded 15 min, and the reduction of the bacterial load followed a different trend for *E. coli*, with respect to *S. aureus* (R_2_ = 0.3652 vs. 0.998).

## 4. Discussion

Non-pharmaceutical interventions, such as hand washing, personal protective equipment (PPE) wearing, isolation, quarantine, personal hygiene, use of disinfectants, and social distancing have been suggested by WHO and the Centers for Disease Control and Prevention (CDC) to contrast SARS-Cov2 dissemination [1,2,3]. Face masks, in particular, have been (and still are) used all over the world, and are considered the most important protective devices during the current COVID-19 pandemic [1,2,3,4,5,6,7]. Wearing face masks in public spaces seems to enforce interpersonal distancing and reduce virus spread by creating a sense of solidarity between people in collectively fighting the pandemic, further increasing compliance [60]. However, there are serious concerns about mask usage, such as the extent of a mask’s filtering efficiency and the potential contamination of both internal and external surfaces. In fact, with continuous use of a mask, breathing and saliva aerosols lead to undesirable microclimatic conditions, due to temperature and humidity, inducing moisture at the face/mask interface. In this microenvironment, filtration efficiency is impaired and bacterial proliferation is favored, imposing a frequent turnover [1,2,3,4,5,6,7,8]. Moreover, even external surfaces are affected, and when contaminated by droplets, undergo the same microbial colonization phenomenon [20,21,22,23,24,25]. Furthermore, given that most face masks are throwaways, their disposals have led to an enormous increase in waste, which, in healthcare environments, is classified as “hazardous with infectious risk” and disposed as biological hazards [19,20,21]. Reusable face masks would be extremely useful, and several methods were proposed for safe reprocessing [9,10,11,12,13,22,61]. Among these, metal and metal oxides, quaternary ammonium or phosphonium groups, antimicrobial polymers, N-halamine compounds, antimicrobial peptides, and natural compounds are reported as antimicrobial strategies (already applied for textiles) [62]. In particular, semiconductors, such as titanium dioxide (TiO_2_), have been tested for their antibacterial efficacy in different matrices, such as water, air, indoor and outdoor environmental surfaces, and fabrics in various materials [35,55]. This compound, in the form of nano- or micrometric particles, generates radical oxygen species when irradiated by a light at the appropriate wavelength, determining the damage of microbial membranes, proteins, and nucleic acids [63,64,65]. Several studies report the use of TiO_2_, both as nano- and microparticles, to confer antimicrobial effects to environmental surfaces and textiles [55,66,67,68]. In regard to the latter application, the technological garments industry is developing synthetic fibers, incorporating TiO_2_ particles to confer different properties, such as antibacterial activity, self-cleaning, and protection from UV. Some of these woven and non-woven fabrics are currently used for commercially available cloth masks. However, the lack of guidelines for testing nanomaterials in their different applications, as well as their elevated heterogeneity, require additional studies, shared protocols, and established regulations [64,65,66,67].

**Figure 8:**
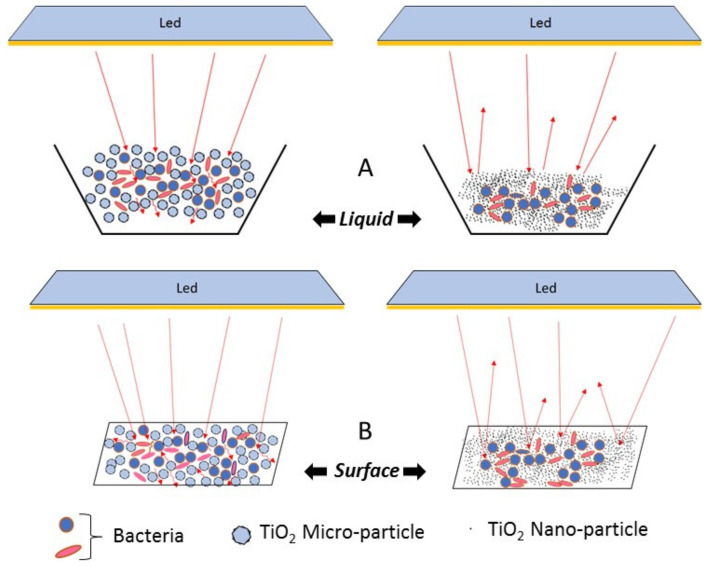
Hypothetical impact of granulometry on the light penetrance and photocatalytic interaction. Putative theoretical model to explain the enhanced effect of micrometric vs. nanometric particles. A reflexing-refraction interference and roughness may favor the interaction between light and microparticles, respectively, in a water suspension or on a coated surface. Specific strategies for fixing particles on a matrix (e.g., chitosan, polysilicon, or others reported in Table 2), may further enhance the light effect. Gram positive and Gram negative bacteria (1 µm) are reported in blue and red, respectively.

This scenario impacts the limits of our study; it does not pose a final “milestone”, but represents an experimental microbiological investigation aimed to present new perspectives. In the first phase of the approach, the antibacterial activity of TiO_2_ nano- and micro-particles was preliminary tested in water under different illumination conditions, following already described protocols [55]. Results show that TiO_2_ NP and MP are both able to reduce the bacterial load within 15 min, when exposed to UV or blue light irradiation. Nanometric TiO_2_ reduced the bacterial load by 50% under blue light and by 40% under UV, while TiO_2_ MP samples—exposed in parallel, under the same conditions—reduced bacterial load by 98–100% both at blue and UV wavelengths. Since an aspecific toxic effect of TiO_2_ on bacteria was reported for nanometric particles, but not at a larger granulometry or for the compound itself, we suppose that the observed findings can be mediated by a photocatalytic action, as further confirmed by data obtained without exposure, in the dark or under different wavelengths (Figure 4). Similar results are reported in the literature, confirming that a toxic effect on bacteria is likely related to the nanometric dimension of the particles, as shown by electron microscopy [62]. One possible explanation for the higher activity of MP vs. NP when exposed to specific wavelengths is reported in Figure 8, proposing a putative geometric role of particles in the interaction with the light, with respect to the action of ROS on microorganisms. Following this theoretical hypothesis, both in a water suspension as well as on a coated surface, the impact of the particulate barrier may differ based on the particle size.

As originally reported by Li and colleagues in 2006, a feasible experimental model to evaluate antimicrobial effects of TiO_2_ and Ag NP on coated mask surfaces is based on artificial contaminations using *E. coli* and *S. aureus* and observing the reduction in viable cells [65]. Following a similar approach, we tested the antibacterial activity of TiO_2_ MP on different face mask materials exposed to 450–455 nm visible blue light (not UV), observing a clear (>99%) reduction in the microbial load after 15 min, already present after 5 min (>80%). These findings suggest a synergic effect due to the combination of TiO_2_, micrometric granulometry, and blue light wavelength exposure. The availability of a photocatalytic (non-chemical) disinfection action driven by non-UV exposure and non-nanometric products may offer a promising alternative for durable antimicrobial applications on masks, overcoming the toxicity related to UV wavelength treatments and nanometric-based technologies. The bactericidal effects of blue light were already reported and well described in the literature, showing their effectiveness on different pathogens, including SARS-CoV-2 [66]. However, the mechanisms of action need to be unraveled. One common hypothesis is that blue light stimulates naturally occurring endogenous photosensitizing chromophores (iron-free porphyrins or/and flavins) in microbial cells, leading to the production of cytotoxic reactive oxygen species (ROS) [60]. However, the visible white light itself, both artificial or sunlight, is known to have some antimicrobial activity. Therefore, we made preliminary random trials on *E. coli* in order to test the effects under common conditions, such as sunlight or available indoor artificial light sources, observing an enhanced antimicrobial effect in the presence of TiO_2_ MP (data not shown). Since the setup of the experiments cannot be conducted fully in the dark, and require the presence of appropriate illumination for the operator handlings, we can suppose that the slight TiO_2_ activity reported by different authors in non-exposed controls may be due to a confounding background, due to the light source used in the hood or in the laboratory to set up the experiments [62,63]. However, further possible mechanisms could play a role in addition to what is determined by light irradiation, supporting a possible antibacterial effect of TiO_2_ itself, independently from light exposure.

The results allow for a series of considerations, supporting a smart approach (in regard to disinfection by light), avoiding the risks related to UV irradiation leading to eye and skin damage [67,68]. The possibility of micrometric TiO_2_ activation by visible spectrum wavelengths can represent a safer, promising, and smart approach [57,68,69,70]. In these experiments, no chemical or physical carriers were used to bind particles to the mask surface; however, several methods are available for coating different matrices, including textiles or other materials used for face masks and PPE (Table 2) [71,72,73,74,75,76,77,78,79,80]. For example, among the different available techniques, the combination of natural polymers, such as chitosan (CS) with inorganic materials, such as titanium dioxide (TiO_2_), to obtain hybrid composites (CS–TiO_2_), seems promising for both increased stability and enhanced antimicrobial properties [81,82].

Furthermore, appropriate and optima fixing of particles would be a key issue for safety, by reducing the risks of inhalation of free TiO_2_ particles or dermal prolonged contact of powder traces with the skin. Otherwise, as for skin contact, TiO_2_ is an active ingredient in many sunscreens, both in the form of micro- and nanoparticles; its use in this specific application is recognized as safe [81] because it does not seem to penetrate the subcutaneous layers [48,49,50]. The opportunity to adopt advanced coating strategies for microparticles would further strengthen the approach, and potential uses in different fields, reducing the free nanometric component and improving the environmental safety. Indeed, risks related to accidental inhalation are strictly correlated to the aerodynamic diameter of the particles, representing a different scenario for nanometric vs. micrometric particles [82].

Further studies are needed to optimize and validate different promising strategies for a photocatalysis-mediated disinfection, in order to test the response of different materials and microorganisms, define the optimal wavelength exposure ranges, and consider alternative solutions to bind particles, including synergic combinations between TiO_2_ and natural or synthetic polymers, opening new perspectives to improve the fixation process of TiO_2_ and enhance the light-induced antimicrobial activity.

The threat posed by the COVID-19 pandemic has strongly modified our lifestyles, with the widespread use of masks, raising the need for new strategies in regard to disinfection and environmental tracing [83,84]. Updating personal protective equipment support safety in the fight against the pathogens should reduce (as much as possible) the impact on the environment. Extending the duration and reuse of masks by photocatalytic disinfection with fixed micrometric TiO_2_ and visible light may represent a promising opportunity to avoid UV frequencies and reduce biocide waste or particles of nanometric granulometry. Advancements in experimental microbiological investigations offer challenging perspectives that require further research to assess sustainability and to transfer scientific knowledge into everyday prevention strategies.

## 5. Conclusions

Disinfecting face masks and personal protective equipment may benefit photocatalytic treatments, presenting an alternative approach to reduce microbial contamination. Mask reprocessing could support safer reuse, extended duration, and reduction of waste. TiO_2_ micrometric particles are effective under visible blue light and could represent a feasible alternative to nanometric granulometry. This approach could also decrease the environmental pollution and toxicity risks due to the nanometric size of particles. The possibility of applying visible wavelengths (and not only traditional UV frequencies) represents an additional safety factor to prolong mask reuse in different, everyday environments, including occupational and recreational settings. In this paper, findings from several experimental investigations were reported on; however, additional research is further needed to clarify mechanisms and implement technologies. Promising perspectives are opening up for photocatalytic treatments in regard to masks and personal protective equipment.

## Figures and Tables

**Table 1:** **Overview of the antimicrobial activity.** Exposure at T_0_ and T_15_ for TiO_2_ microparticle film in cloth face masks, surgical face masks, and FFP2 masks after exposition to blue light. TiO_2_ treated and untreated masks were contaminated with *E. coli* and *S. aureus*.

***E. coli***	***Cloth Face Mask***		***Surgical Face Mask***		***FFP2***	
***Contact time***	***Coated***	***Uncoated***	***Coated***	***Uncoated***	***Coated***	***Uncoated***
*Time 0*	5.0 ± 1.6 × 10^3^	5.0 ± 1 × 10^3^	5.0 ± 1.8 × 10^3^	5.0 ± 1.8 × 10^3^	9 ± 3 × 10^3^	9 ± 3 × 10^3^
*Time 15’*	0.0	2 ± 3 × 10^2^	3 ± 3 × 10^2^	1 × 10^3^	2 ± 4 × 10^2^	9 ± 1 × 10^2^
*% of reduction*	100.0	96.0	97.0	80.0	98.0	90.0
***S. aureus***	***Cloth Face Mask***		***Surgical Face Mask***		***FFP2***	
***Contact time***	***Coated***	***Uncoated***	***Coated***	***Uncoated***	***Coated***	***Uncoated***
*Time 0*	5.0 ± 1.8 × 10^3^	5.0 ± 1.8 × 10^3^	1.3 ± 1.0 × 10^3^	8 ± 1 × 10^2^	9 ± 3 × 10^3^	9 ± 3 × 10^3^
*Time 15’*	0.0	5 ± 3 × 10^2^	1.6 ± 1.0 × 10^2^	5 ± 1 × 10^2^	0.0	3 ± 1 × 10^2^
*% of reduction*	100.0	80.0	88.0	62.5	100.0	98.0

**Table 2:** Overview of the different (available) methods for TiO_2_ coating surfaces from different matrices, including textiles.

Application Method	Description	Reference
Immersion, pad-dry cure	Fabric immersion in a TiO_2_ suspension, following by a drying step at 80 °C for 5 min, cured at 180 °C for 3 min. The process is then repeated, then washed with deionized water at 60 °C for 5 min and dried at 60 °C again.	[71,72]
Electrospray	A suspension of TiO_2_ is dissolved in a polar solvent, nebulized at atmospheric pressure inside the ionization chamber through a needle held at a high electric potential.	[73]
Dip coating, spray coating	Deposition of a wet liquid film by immersion of the fabric into a solution containing hydrolysable metal compounds (or readily formed particles) and its withdrawal at a constant speed into an atmosphere containing water vapor.	[74,75]
Irradiation under UV-A	Textile immersion in a suspension of TiO_2_, then squeezed and immediately irradiated under UVA 100 W lamp for 1 h. Finally, the coated fabric is dried at 130 °C for 5 min and cured at 150 °C for 3 min.	[76]
Plasma treatment with direct current magnetron sputtering	Plasma treatment makes fabrics and technical felts hydrophilic and allow wettability with aqueous processing substances in particular stages of the process, such as dyeing, printing, or textile finishing. Then, samples are impregnated with TiO_2_.	[77]
Electrospinning	Electrospinning is a process by which a polymer in solution or spindle can be spun into small diameter fibers, thanks to a high potential electric field. Cellulose acetate is used as the core phase, which, after a deacetylation step, becomes cellulose. A dispersion of TiO_2_ particles is used as the sheath phase to disperse titania nanoparticles along the fiber outer surfaces.	[78]
Some polymers and proteins functionalized with TiO_2_ particles	By different coating techniques, such as a dip–pad–dry cure process, a chitosan–titanium dioxide (CS–TiO_2_) composite or CS-TiO_2_ with citric acid can be applied into a cotton matrix.	[79,80]

## Data Availability

Not applicable.
